# Protocol of Human Primordial Germ Cell-like Cell Generation from Pluripotent Stem Cells in 2D and 3D Culture Systems

**DOI:** 10.3390/biology15161436

**Published:** 2026-08-20

**Authors:** Vepa K. Abdyev, Polina I. Sirotkina, Evgeniia D. Erofeeva, Mariia A. Erokhina, Nikita Y. Grudinin, Ekaterina A. Vorotelyak, Andrey V. Vasiliev

**Affiliations:** 1 Koltzov Institute of Developmental Biology, Russian Academy of Sciences, ul. Vavilova, 26, 119334 Moscow, Russia; erofeeva.zhenya@gmail.com (E.D.E.); maryckx1994@mail.ru (M.A.E.); nikitagru2002@gmail.com (N.Y.G.); vorotelyak@yandex.ru (E.A.V.); 113162@bk.ru (A.V.V.); 2 Department of Biology, Lomonosov Moscow State University, 1-12 Leninskie Gory, 119991 Moscow, Russia; sirotkinapolina@gmail.com

**Keywords:** primordial germ cells, hPGCLCs, gametogenesis, PSCs, *NANOS3*, *DAZL*, *NANOG*

## Abstract

Because human infertility can arise from genetic, molecular, cellular, anatomical, and endocrine abnormalities, in vitro gametogenesis has become an important area of reproductive research. New developing technology involving the generation of human primordial germ cell-like cells (hPGCLCs) from induced pluripotent stem cells (hIPSCs) might assist with understanding early germ cell development (specification, migration, gametogenesis, and epigenetic reconstitution), as well as provide a solution for infertility and hereditary disorders. Given that human primordial germ cells (PGCs) are still not well characterized at a molecular level, we present a practical workflow to robustly and efficiently induce hPGCLCs from human pluripotent stem cells (hPSCs) XX and XY cell lines. This protocol describes the hPGCLC specification of hPSCs through sequential induction with Activin A for 2 days and BMP4 for 6 days in 2D and 3D culture systems. Induction of hPSCs into hPGCLCs demonstrated expression of early primordial germ cell markers, including PRDM1, NANOS3, DAZL, STELLA, SOX17, SSEA1, and cKIT, on the 8th day of hPGCLC generation. We described the protocol for generating early hPGCLCs, which provides an opportunity for further investigations into maturation into late germ cells and a chance to overcome a meiotic block to obtain haploid gametes in vitro.

## Simple Summary

Human in vitro gametogenesis is the focus of researchers, as pluripotent stem cell derived human primordial germ cell-like cells (hPGCLCs) could not complete the meiotic division. Therefore, generating early hPGCLCs, which provides an opportunity for further investigations to overcome a meiotic block, is a cue of success in deriving haploid gametes in vitro. Thus, we described a protocol for hPGCLC specification of human pluripotent stem cells (hPSCs) through sequential induction with Activin A for 2 days and BMP4 for 6 days in 2D and 3D culture systems. Induction of hPSCs into hPGCLCs demonstrated expression of early primordial germ cell markers, including PRDM1, NANOS3, DAZL, STELLA, SOX17, SSEA1, and cKIT, on the 8th day of hPGCLC generation.

## 1. Introduction

Primordial germ cells (PGCs) are progenitor cells of haploid gametes, whose fusion is resolved through passage of genetic and epigenetic information to the next generation. However, despite the successful application of various protocols in generating human primordial germ cell-like cells (hPGCLCs) in vitro [1], a molecular aspect of hPGCs needs further characterization [2], and although hPGCLCs gained expression of later (peri- and post-migratory) markers, successful completion of meiosis has not yet been achieved [2]. Therefore, further elucidation of hPGCLC specification and maturation remains a focus of interest to investigate the molecular, epigenetic, and functional aspects of human gametogenesis in vitro.

A proper, reproducible, and efficient in vitro model of hPGC differentiation from human embryonic stem cells (hESCs) and human induced pluripotent stem cells (hiPSCs) will be used to scrutinize human gametogenesis. Therefore, to precisely understand the mechanisms of specification and formation of hPGCs, it is important to standardize an in vitro hPGCLC derivation protocol from human pluripotent stem cells (hPSCs) that is reproducible, fully controllable, well described, and efficient. In vitro generation of human germ cells began with spontaneous differentiation from hESCs [3,4,5] and has since developed into fully described hPGCLC derivation protocols, which have been systematically analyzed and elaborately reviewed in recently published reviews [1,2].

**Figure 1:**
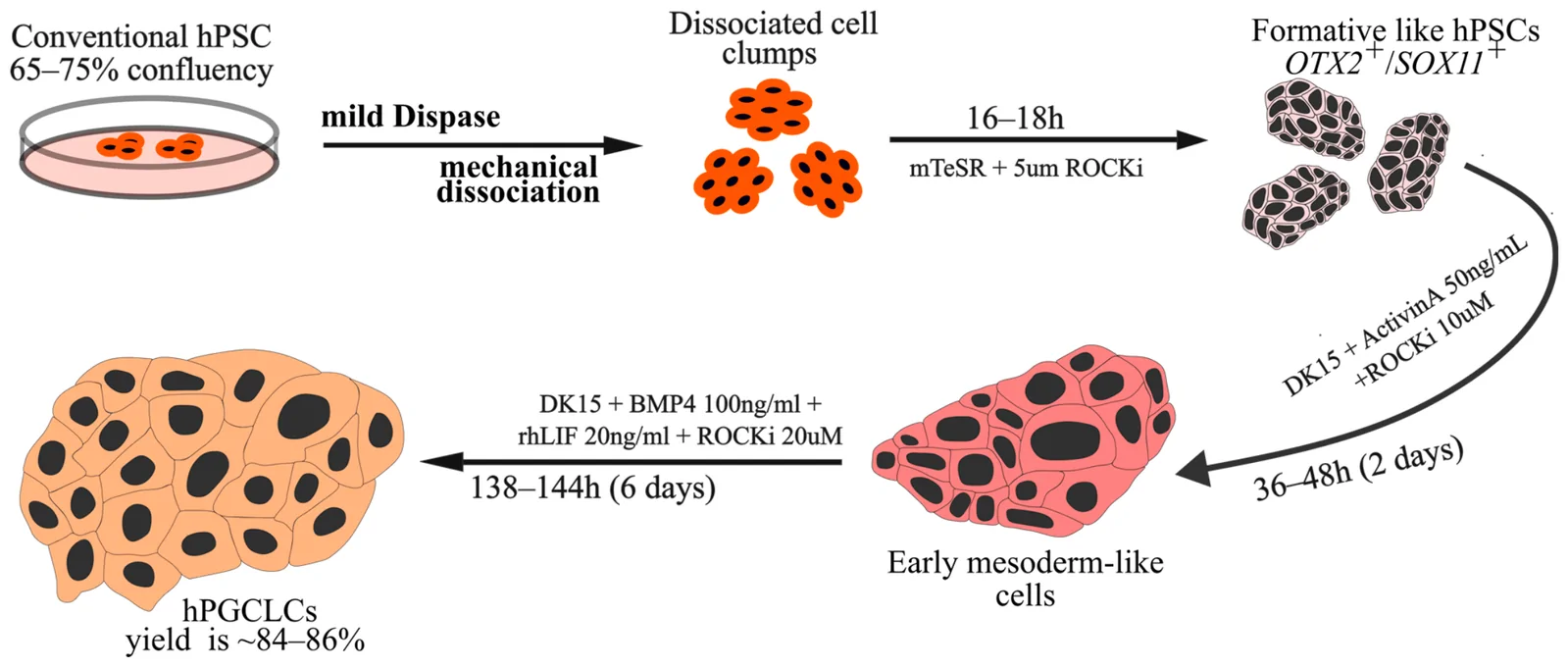
Schematic illustration of hPGCLC derivation from hPSCs.

To develop hPGCLCs from hPSCs that can be used for further investigations to overcome meiotic block in vitro [6,7] in haploid cell derivation, we applied a sequential induction protocol via the early mesodermal push of female and male hPSCs (Figure 1). The incipient mesodermal transition applying Activin A [8,9] sequestered cell morphological alterations, like an increase in the cytoplasm-to-nucleus ratio with upregulation of *PRDM14*, *KLF4*, *NANOG* and other master pluripotency genes like *OCT4* and *DAZL*, and coincided with the activation of early germ cell key regulators such as *PRDM1*, *NANOS3*, *SSEA1* and *cKIT* [7,10].

Further induction with BMP4 of early mesoderm-like cells, which demonstrated resonance with early germ cell marker-gene activation, directed their expression profiles toward early (PRDM1 and NANOS3) and late (VASA and DAZL) germ cell markers [7]. We demonstrated that SOX17, an upstream regulator involved in germ cell differentiation [11], PRDM1, and NANOS3 were observed in both the cytoplasm and the nucleus of SSEA1^+^ cells. Cells were stained for STELLA, which localizes to the perinuclear region of hPGCLCs, and for DAZL, which is co-expressed with SSEA1 in hPGCLCs [7].

The meiotic activation of DAZL^+^ and VASA^+^ late hPGCLCs using retinoic acid (RA) revealed meiotic initiation by expressing the marker *STRA8* [7]. Furthermore, upon meiotic induction, upregulation of the late germ cell markers *VASA*, *KLF4*, *PIWIL4*, and the mid-pachytene-demarking gene *PIWIL1* was observed [7]. As a result of the applied differentiation protocol, the *XIST* gene was altered in expression in generated hPGCLCs, which confirmed the possible activity of both X chromosomes in XX cells. In the nucleus of the cells, the meiotic marker SCP3 demonstrated a pointed signal [7]. These results demonstrated that the derived hPGCLCs from hPSCs XX and/or XY using sequential induction display several characteristics of PGCs.

## 2. Materials and Methods

### 2.1. Reagent Setup (Medium)

#### 2.1.1. FACS Buffer

Dilute 450 μL of FBS (Table 1) in 14.5 mL of 1× PBS to obtain a PBS, 3% (vol/vol) FBS solution. The solution can be stored at 4 °C for 5 days.

#### 2.1.2. FACS Primary Antibody Solution

In 99 μL of FACS buffer, dilute 1 μL of mouse monoclonal anti-SSEA1 antibody (1:100) (Table 2). Keep the solution on ice and protected from light until use. Using no more than 1,000,000 unstained starting cells per 100 μL of FACS primary antibody solution is recommended.

#### 2.1.3. Blocking Buffer

To obtain permeabilization buffer, mix prewarmed 10 mL of PBS, 0.1% (vol/vol) Triton X-100, and 0.02% Tween20. Dilute 500 μL of FBS and/or donkey serum in 9.5 mL of permeabilization buffer. The solution can be stored at 4 °C for ≤2 days.

#### 2.1.4. Immunofluorescence Primary Antibody Solution

In 100 μL of blocking buffer, dilute 1 μL of primary antibody (Table 2). Freshly prepare the solutions on the day of the experiment. Keep the solution on ice until required. This protocol can be adjusted to other primary antibodies of interest.

#### 2.1.5. Immunofluorescence Secondary Antibody Solution

In 999 μL of blocking buffer, dilute 1 μL of Alexa Fluor 488 donkey anti-mouse (1:1000) and 1 μL of TexasRed donkey anti-rabbit (1:1000). Prepare the solutions freshly on the day of the experiment. Keep the solution on ice and protected from light until required. This protocol can be adjusted to other secondary antibodies of interest.

#### 2.1.6. hPGCLC Basal Medium—DK15

The basal medium consists of DMEM/F12, 15% KSR, 0.1 mM NEAA, 2 mM GlutaMax, 1 mM Sodium Pyruvate, and 0.1 mM β-Mercaptoethanol (Table 1).

Troubleshooting: KSR is a batch-dependent reagent, so refer to the manufacturer’s documentation. Our experience of using KSR (10828010, Gibco) has shown no deviations in applications.

#### 2.1.7. DK15 + rhActivinA

Basal medium (DK15) is supplemented with 50 ng/mL rhActivin A (Activin A) and 10 μM ROCKi.

#### 2.1.8. hPGCLC Induction Medium

DK15 is supplemented with 100 ng/mL rhBMP4, 20 ng/mL rhLIF, and 20 μM ROCKi.

The female hESC line was deposited in Cellosaurus as hESMK05 (CVCL_C647) [12] and was kindly gifted by Lagarkova M.A.

The female hIPSC line (KYOU-DXR0109B Human Induced Pluripotent Stem Cells [201B7] (ATCC^®^ ACS-1023™)) was purchased from the American Type Culture Collection (ATCC).

The hPSC lines were cultured in mTESR1 complete medium and accepted as primed PSCs. The protocol was applied, and results were obtained in both lines. The presented results are shown in the Figures.

Troubleshooting: Cell lines should be periodically checked for karyotype integrity and mycoplasma infection before the protocol application.

### 2.2. Estimated Overall Workflow

If Parts A and B are performed sequentially, we have the following:

- Part A: ~17 h (overnight).
- Part B: ~8 days.
- Total: Approximately 9 days from hPSC passaging to hPGCLC analysis.

### 2.3. Procedure

#### 2.3.1. Part A—hPSCs Maintenance

1. Coat the needed number of wells of a 6-well plate (Table 3) with 1× Matrigel before cell passage and leave for 15–45 min for polymerization (Table 4 and Table 5) in a cell culture incubator.

Troubleshooting: Matrigel is a batch-dependent reagent, so refer to the manufacturer’s documentation.

2. Collect the old culture medium from the wells of hPSC into the centrifuge tube for further use as a conditioned medium for enzymatic dilution and/or inactivation. Centrifuge old conditioned mTeSR1 medium from the culture at 300× *g* for 3 min.

Troubleshooting: Avoid using hPSC culture with more than 5% spontaneous differentiation. This will affect the hPGCLC generation yield. Do not use the culture with more than 75% confluency.

3. Add 1 mL of 1× PBS at room temperature into the well with cells, wash by gently shaking the plate, and discard.
4. Add 500 uL of prewarmed 37 °C Dispase solution to the well and incubate in a 5% CO_2_, 5% O_2_ humidified incubator at 37 °C for 3–4 min (Table 5). The dissociation time may vary, and cells should be monitored under the microscope to determine the optimal time.

**Alternative:** Add 500 uL of prewarmed TrypLE Express/Accutase to cells and place the culture plate in a 5% CO_2_, 5% O_2_ humidified incubator at 37 °C for 3 min to dissociate PSC colonies into single cells and use for embryoid body (EB) formation.

5. In the cell culture treated with Dispase, the edges of colonies should rise and wrap toward the center. The PSC colonies should rise at the edges but not wrap toward the center. At this moment, gently pipette the Dispase-treated PSC culture 5–10 times with Dispase, and due to the intensive flow of the stream, the colonies should be detached. At this moment, add 2 mL of conditioned medium (step 2), and again gently pipette up and down 10–15 times with a 1000 µL pipette to dissociate colonies/large clumps into smaller clumps.

Alternative: In the case of TrypLE Express, add 2 mL of conditioned medium and gently pipette up and down to detach the cells and obtain a single-cell suspension.

6. Collect the cell suspension and transfer it into a 15 mL centrifuge tube and centrifuge at 300× *g* for 3 min.
7. Remove the supernatant. Resuspend the cell pellet in 1000 μL of mTESR1 complete medium supplemented with 5 μM ROCKi.
8. The hPSC suspension from Step 7 should be diluted at a proportion of 1:5 to 1:10 by adding 100–300 μL of the cell suspension to 1.5 mL of mTESR1 complete medium supplemented with 5 μM ROCKi in a 1.5 mL tube.
9. Remove the Matrigel-coating solution from the pre-coated well and add 1.5 mL of the diluted cell suspension prepared at Step 8.
10. Gently rotate the plate back and forth, and left and right, to ensure a homogeneous and even distribution of cells and cell clumps across the Matrigel-coated culture surface, following a cross-orientation. Repeat this step several times.
11. Transfer the culture plate for further inoculation and cell clump adhesion into a 5% CO_2_ and 5% O_2_ humidified incubator at 37 °C for 16–18 h.
12. On the following day, proceed with the hPGCLC differentiation protocol

#### 2.3.2. Part B—hPGCLC Differentiation from Female Human ESCs/IPSCs in an Adhesive 2D System

**Figure 2:**
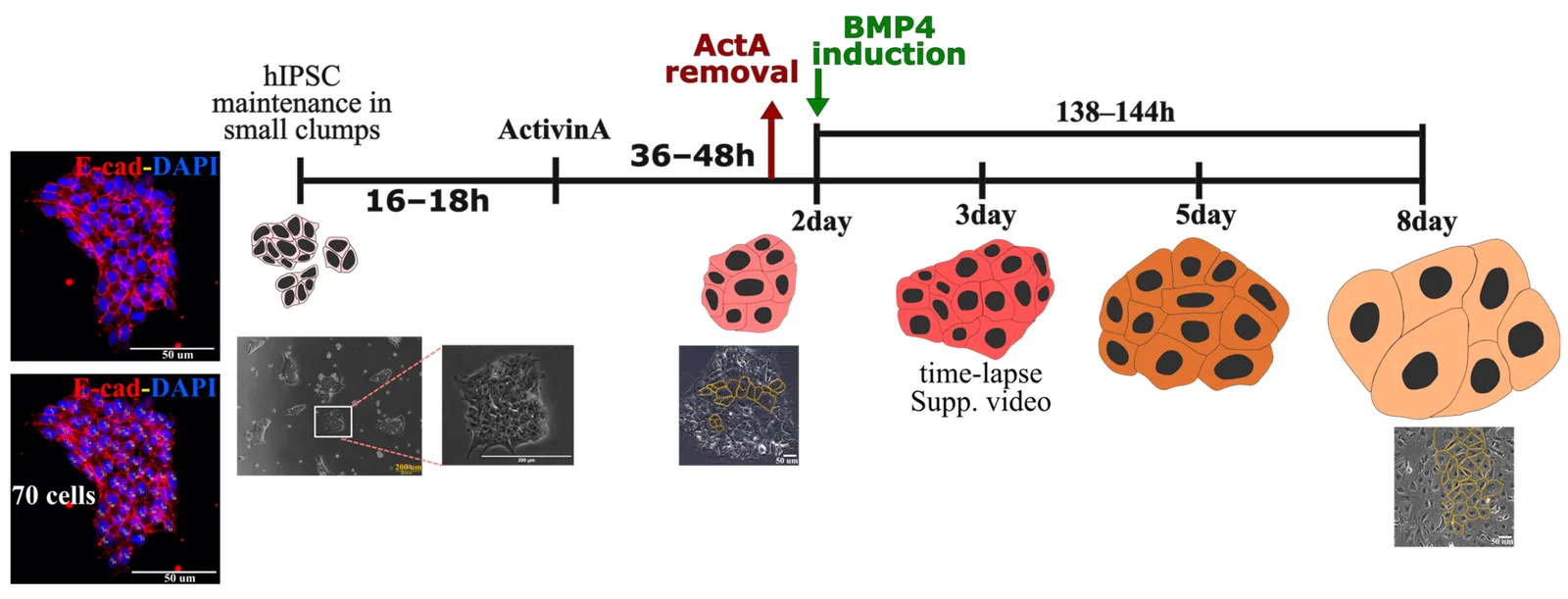
Schematic illustration of timeline and cell morphology changes in differentiating cells. Phase contrast images of colonies of hIPSCs in small clumps (capture of 1 colony), early mesoderm-like cells (on the 2nd day; illustrative pink cells), and hPGCLCs (on the 8th day; illustrative light brown cells). Dashed lines encircle cell morphology. A fluorescent image of an hIPSC colony consisting of 70 cells was stained for E-cadherin (red), and cell nuclei were counterstained with DAPI (blue). Scale bars are 50 μm and 200 μm.

1. After maintenance and achieving small colonies consisting of 50–200 cells at Part A—Step 11 on the following day, discard the old medium and induce the cells with 50 ng/mL Activin A supplemented with 10 μM ROCKi for 36–42 h (Figure 2). Daily medium changes should be performed.

Troubleshooting: Avoid culturing hPSCs in small clumps after inoculation for more than 16–18 h to achieve the needed colony size. This will affect differentiation yield, pushing cells toward spontaneous differentiation. The colony sizes during hPSC inoculation should be empirically determined. Large colonies will have a dense center that will maintain pluripotency to some extent. Small colony clumps will differentiate into more mesoderm-like cells.

2. After 2 days of Activin A induction, body-treat the culture with secondary antibodies (1:1000) in hPGCLC induction medium for 1.5 h at 37 °C with 5% O2 and 5% CO_2_. After washing the attached cell culture with DMEM/F12 twice, wash the cells with PBS without Ca^2+^/Mg^2+^ and treat with Dispase/Accutase for 3–7 min at 37 °C. The cells should be detached into a dissociated single-cell condition by gentle pipetting. Add 5 mL of FACS buffer and centrifuge at 300× *g* for 3–5 min. Discard the supernatant and resuspend the pellet in 1 mL of FACS buffer. Run the cell suspension through a cell strainer for flow cytometry. The sample is ready for analysis and sorting.

Troubleshooting: Controls for FACS analysis should be considered: control group minus primary antibodies; control group plus primary antibodies but minus secondary antibodies; and control group of cells that are negative for SSEA1.

#### 2.3.3. Part C—EB Generation and hPGCLC Differentiation in EB

1. Collect the old culture medium from the well of hPSCs into the centrifuge tube to further use as a conditioned medium for enzymatic dilution and/or inactivation. Centrifuge the old conditioned mTeSR1 medium from the culture at 300× *g* for 3 min.
2. Add 1 mL of 1× PBS at room temperature into the well with cells and rinse gently by shaking the plate, then discard.
3. Add 500 µL of prewarmed TrypLE Express/Accutase to the cells and place the culture plate in a 5% CO_2_, 5% O_2_ humidified incubator at 37 °C for 3 min.
4. Add 2 mL of conditioned medium (Part C—Step 1) and gently pipette up and down to detach the cells to obtain a single-cell suspension. Using an automated cell counter, determine cell concentration and viability.
5. EB generation can be achieved and continued with the AggreWell kit. To generate EBs in hanging drops, collect the cell suspension, transfer it into a 15 mL centrifuge tube, and centrifuge at 300× *g* for 3 min.
6. Remove the supernatant. Resuspend the cell pellet in 1000 μL of mTESR1 complete medium supplemented with 5 μM ROCKi.
7. To achieve 4000 cells/EB, calculate the cell concentration and dilute for EB generation. The cell suspension of 4 × 10^5^ cells for 100 EBs should be prepared in 1000 μL fresh mTESR1 complete medium supplemented with 10 μM ROCKi. Perform gentle pipetting to achieve even distribution of single cells. Filter out cell clumps using a 40 μm cell strainer.
8. Apply 10 μL of cell suspension containing 4000 cells in each droplet on the inner side of the cover lid of a 100 mm-diameter Petri dish. From 1 mL of cell suspension, 100 droplets should be generated. After droplet formation, gently and quickly turn the Petri lid with droplets upside down and close the Petri dish filled with 5 mL of DMEM/F12 with the lid containing droplets.
9. Transfer the hanging droplets for further aggregation into a 5% CO_2_ and 5% O_2_ humidified incubator at 37 °C for 16–18 h.
10. Perform EB differentiation on ultra-low-attachment Petri dishes after generation of EBs with AggreWell kit and/or hanging drops. Transfer generated EBs from AggreWell plates/hanging drops to ultra-low-attachment dishes.
11. To transfer EBs in hanging droplets, gently open the cover lid of the Petri dish with hanging droplets and fix at 45 ° with a 1000 µL pipette. Flush using DMEM/F12 droplets with EBs. Collect the flushed EBs in suspension from the edge of the cover lid of the Petri dish and flush gently through a 40 μm cell strainer to collect EBs. The retention of EBs on the mesh should occur on the bottom of the outer side of the cell strainer to ensure collection and easy transfer of collected EBs. After flushing, the EBs should remain on the bottom surface of the cell strainer. Invert the cell strainer with EBs and flush with DK15 + rhActivinA medium from the inner bottom wall toward the ultra-low-attachment Petri dish.
12. Induce EBs with 50 ng/mL Activin A and 10 µM ROCKi in DK15 for 36–42 h (2 days) (Figure 2). Daily medium change is required.

Troubleshooting: To change the medium, concentrate the discarded EBs at the center of the plate, and from the corners of the plate, start to discard the medium by gently aspirating with a pipette.

13. The Activin A-induced EBs are triggered with 100 ng/mL BMP4 and maintained for 138–144 h (6 days) in DK15 with 100 ng/mL BMP4, 20 ng/mL rhLIF, and 20 uM ROCKi. Daily medium change is required.

## 3. Expected Results

The 65–75% confluency of hPSCs is an important point because cells are in an active cell cycle and self-renewal state [13]. Therefore, the combination of mild enzymatic and mechanical cell detachment of hPSC colonies into small clumps by applying Dispase and further pipetting (mechanical dissociation) may stimulate cells to shift the pluripotency state toward formative PSCs. Since the enzymatic-based single-cell passaging demonstrated alterations in colony morphology and cell nucleus enlargement, accompanied by downregulation of pluripotency markers [14], we have combined mild enzymatic dissociation with mechanical tuning of the size of cell clumps for further hPGCLC induction. Obtained small cell clumps preserve E-cadherin-mediated cell–cell adhesion, which was shown to be necessary for the maintenance of pluripotency in hIPSCs through the balance of abundance and/or composition of adhesion complexes [15]. E-cadherin expression supports the cuboidal morphology of cells, forming cobblestone colonies.

**Figure 3:**
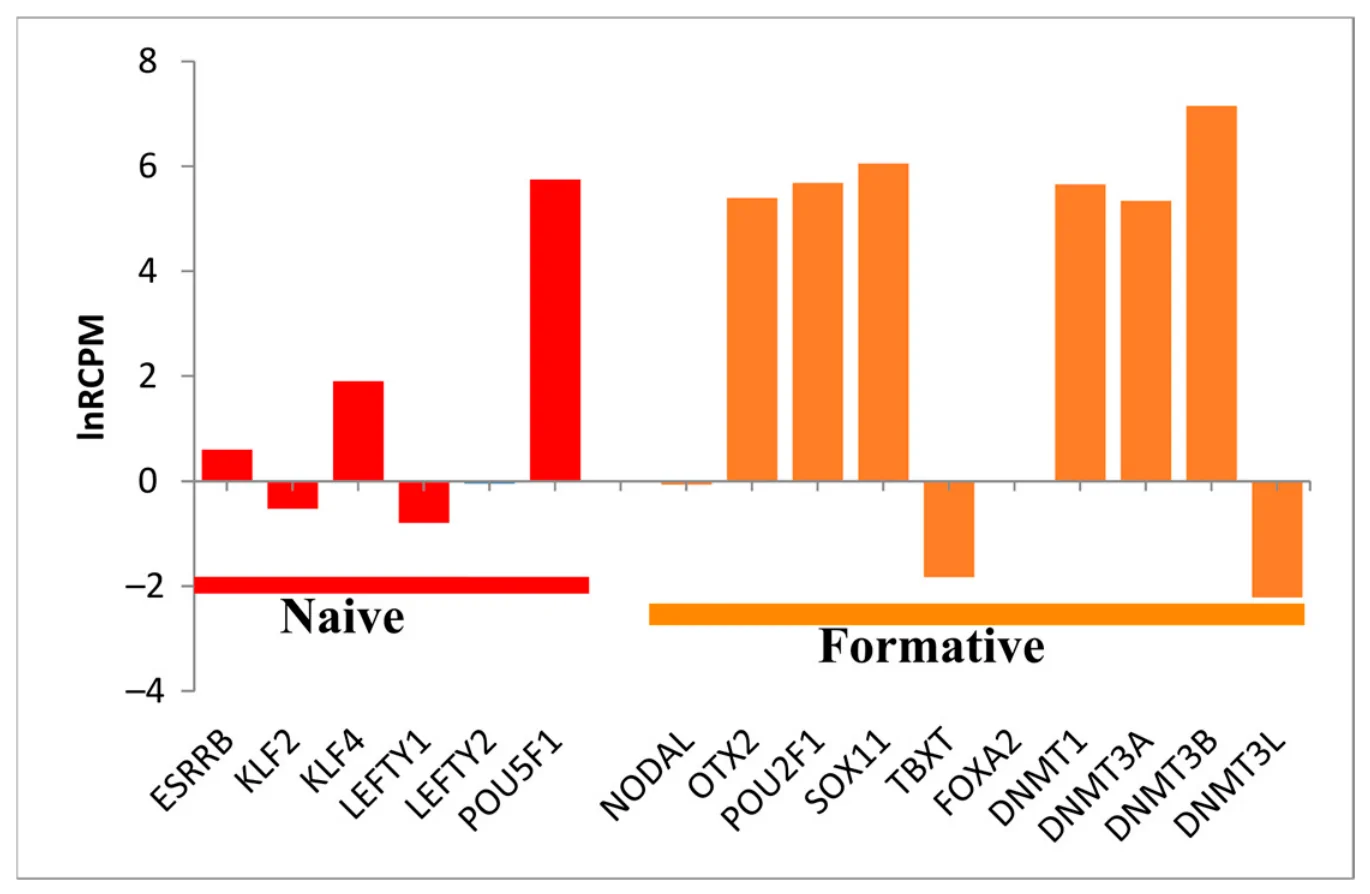
Results of the hIPSC transcriptome analysis of genes characterizing the state of pluripotency. The graph shows the genes of naive and formative PSCs. The values are provided as lnRCPM. The Y-axis represents the logarithmic values of the mRNA copies in the transcriptome. The X-axis represents the genes of interest. lnRCPM stands for the logarithm of Read Counts Per Million. In bulk RNA-seq analysis, two samples (n = 2) were included. Genes that received an FDR below 0.05 from this comparison were removed for clustering analysis to reduce batch effects. Genes with minimal expression (below 3.0 RCPM in all samples) were also removed for clustering purposes.

In contrast, depletion of E-cadherin between cells in hIPSC colonies coincides with cleaved Caspase-3 and/or strong Rac1 expression in the cells [16]. In human PSCs, E-cadherin helps to maintain OCT4/NANOG-positive undifferentiated cells, improves survival after passaging, supports clonal expansion, and can substitute for some matrix-dependent cues when presented as an E-cadherin-based culture surface [17], while single-cell dissociation of hESCs at low plating density results in E-cadherin and alkaline phosphatase downregulation leading to apoptosis or differentiation [18]. At the same time, preserving cells as small E-cadherin-positive aggregates may create a permissive epithelial context for subsequent hPGCLC induction, because human PGCLC protocols typically require transition through an epiblast-like/iMeLC state and aggregation-based differentiation, where controlled cell–cell communication and BMP responsiveness are essential [6]. In addition, detailed analysis of our transcriptomic data (Abdyyev et al., 2020) revealed upregulation of *OTX2*, *SOX11*, *DNMT3A/B*, and *DNMT1* gene markers (Figure 3) of formative hIPSCs [7,19]. Thus, we avoided excessive single-cell stress while maintaining pluripotency-supporting adhesion and preparing cells for efficient, germline-competent formative/post-implantation-like differentiation.

### hPGCLC Induction

**Figure 4:**
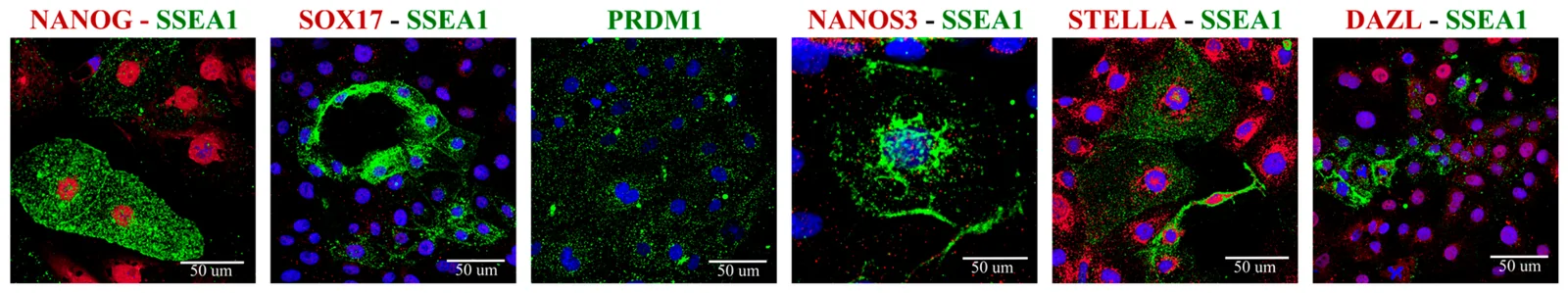
hPGCLC characterization in an adhesive 2D culturing system. 138–144 h (6 days) of BMP4-induced PSCs differentiated into hPGCLCs in an adhesive 2D culturing system on Matrigel. NANOG, SOX17, NANOS3, STELLA, and DAZL are represented in red. PRDM1 (green) was stained in clusters in the cytoplasm at the nuclear periphery. NANOG, SOX17, and NANOS3 were localized in the nucleus and cytoplasm. STELLA and DAZL were stained in clusters at the nuclear periphery and in the cytoplasm. Cell-surface signals of SSEA1 were represented by a green signal. Nuclei were stained with DAPI (blue). Scale bar: 50 μm.

**Figure 5:**
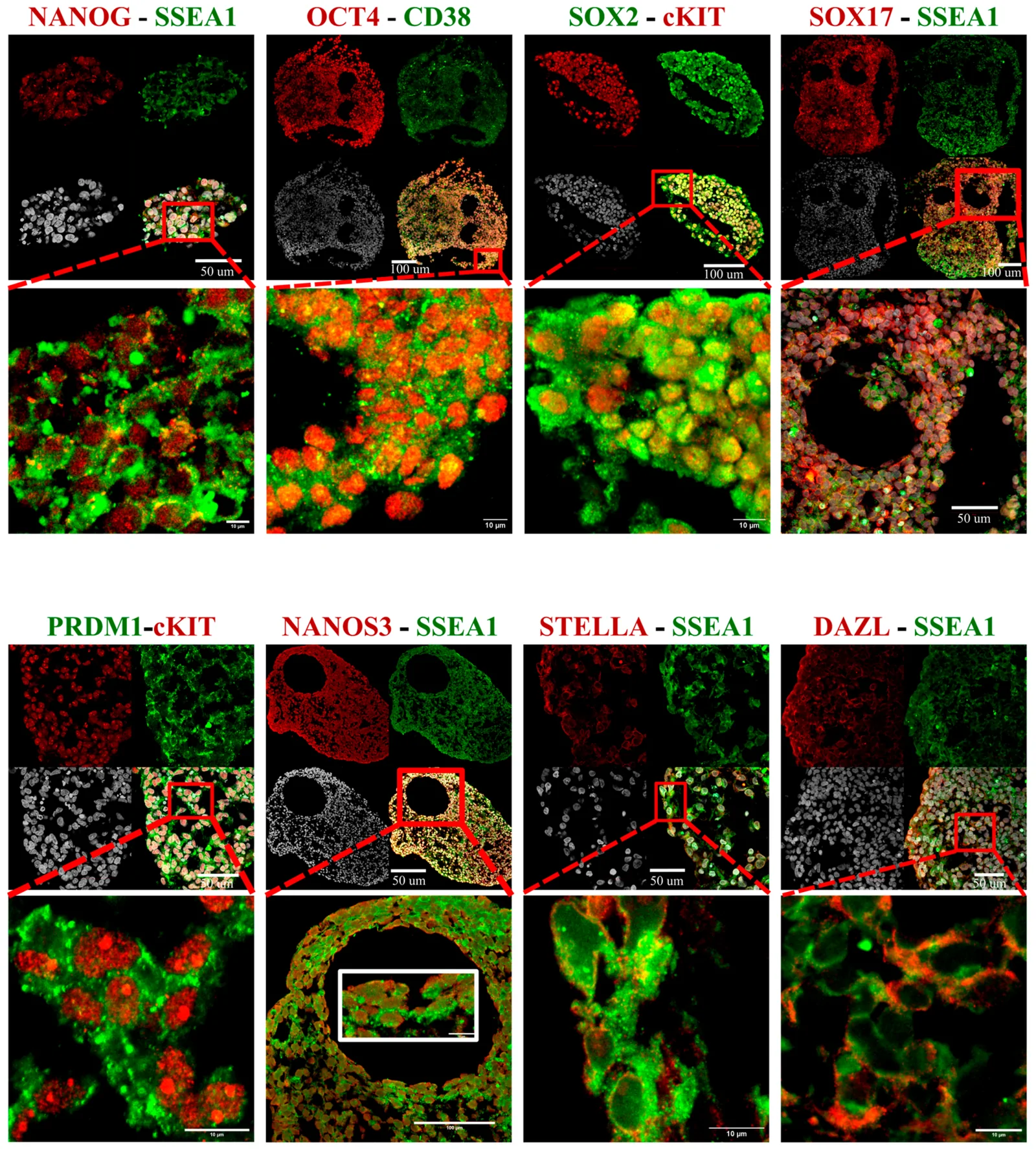
Characterization of hPGCLCs in a 3D culturing system. Immunofluorescent staining for pluripotency markers OCT4 (red), SOX2 (green), and NANOG (red), and germ cell markers SOX17 (red), DAZL (red), STELLA (red), PRDM1 (green), NANOS3 (red), SSEA1, and cKIT (red) of 6 days; BMP4 induced EBs after 2 days of Activin A induction (8 days in total). White box capture illustrated the SSEA1 (green) and NANOS3 (red) staining. Nuclei were stained with DAPI (blue). Cell-surface signals of SSEA1 (green) and CD38 (green). Scale bars: 10 μm, 50 μm, and 100 μm.

An early mesodermal transition using 2d Activin A induction of hPSCs brought morphological flattening of the cells (Figure 2) due to an increase in the cytoplasm-to-nucleus ratio. On the 8th day of the protocol for hPGCLC induction in an adhesive culture system and 3D culture, it was revealed that NANOG remained at high levels in the hPGCLC nucleus (Figure 4), and *NANOG* expression was unchanged [7]. Although *NANOG* expression was quite dynamic, it was upregulated at first but downregulated upon BMP4 induction [7,10]. NANOG presence in hPGCLCs was shown in an embryonic-like culture [20] and in human hindgut organoids [21]. The upregulation of *NANOG* and other master pluripotency genes like *OCT4* and *DAZL* coincided with the activation of early germ cell key regulators such as *PRDM1*, *NANOS3*, *SSEA1*, and *cKIT* at 2d of Activin A induction [7]. It maintained their activity at the 6th day of BMP4 induction in hPGCLCs in a 2D culture system and in a 3D system (Figure 4 and Figure 5). BMP4 induced changes in cell morphology and increased motility in adhesive culture (Supplementary Video S1); this is characteristic of typical migrating primordial germ cells in vivo [22]. After 6 days of BMP4 induction in the 2D culture system and 3D culture of early nascent mesodermal cells, immunofluorescent analysis revealed the coincidence of SOX17 with the surface marker of hPGCs, SSEA1, which also co-stained with the early germ cell markers NANOS3 and STELLA (Figure 4 and Figure 5). DAZL, the late PGC marker, accompanies hPGC maturation and identity (Figure 4 and Figure 5). In addition, *DAZL* expression [7] and its protein presence demarcate the continuous maturation of hPGCLCs. The maturation of hPGCLCs in vitro has not been broadly investigated; the results of *DAZL* and *SSEA1* co-expression are not conflicting with their maturation, since maturation is not strictly bordered but a continuous process.

BMP4 induction of EBs revealed the expression of early primordial germ cell markers, including PRDM1, NANOS3, STELLA, and cKIT (Figure 5), on the 8th day of hPGCLC generation (Figure 2). *cKIT* [23] and CD38 markers of hPGCLCs [11] demonstrated positive signals in cells of EBs (Figure 5). The results presented in this paper support hPGCLC induction from hPSCs. This protocol can easily be applied in in vitro studies to advance our understanding of early human germ cell developmental processes. The protocol generates hPGCLCs enriched for cells expressing multiple early germ-cell-associated markers under both adherent and EB-based conditions.

## 4. Discussion

The differentiation protocol we developed for hIPSCs and hESCs into PGC-like cells demonstrated a yield of 86.07% and 84.56% hPGCLCs (SSEA1^+^), respectively [7], representing a high yield of differentiated cells. We have developed a strategy and principle of mathematical set intersection, which was demonstrated in immunofluorescent images. Figure 4 and Figure 5 demonstrate the co-staining of most PGC markers, NANOG, SOX17, NANOS3, DAZL, and STELLA, with SSEA1. The results of Figure 4 and Figure 5 were consistent and supported by separately stained samples for PGC markers, in which SSEA1 was used as an intersection set for all PGC markers, so that the purpose of using SSEA1 is to obtain early hPGCLCs through live-cell FAC sorting and to use pure SSEA1^+^ cells for further maturation and gametogenesis (meiotic division). Therefore, we have consolidated SSEA1 as a solely non-invasive reporter that represents all early PGC markers, so that sorting of SSEA1^+^ cells ensures that the obtained cells are hPGCLCs.

The limitation of the protocol can be related to hPSC homogeneity, as the culture should avoid spontaneous differentiation above 5%. Another limitation is the pluripotency state, which is not chemically defined but depends on mechanical manipulations. Despite these possible limitations, repetitive hPSC differentiation in 2D/3D culture systems resulted in hPGCLC generation. In addition, hPGCLCs derived from 2D culture systems poorly progress through maturation and inadequately respond to meiotic induction.

## 5. Conclusions

The researchers hope that studying the processes of PGC development in vitro from hIPSCs will help us to understand the mechanisms of human PGC specification and differentiation and lead to the modulation of adequate models of infertility, congenital anomalies, etc. On the other hand, the production of human gonocytes in vitro could be a new direction of assisted reproductive technologies (ART). The prospects of research on hPGCLCs and their derivatives obtained in vitro include their use in pharmacology to create test systems that study the effect of drugs on human germ cells, as well as the possibility of studying the molecular pathogenesis of diseases such as germ cell tumors, aneuploidy, and sex chromosome abnormalities. hPGCLCs might be a promising model for human genome editing of hereditary diseases. To summarize, maintaining human PSCs and differentiating them into hPGCLCs in vitro is a promising field of biomedicine, the development of which will lead to a breakthrough in understanding the mechanisms of germ cell maturation and in vitro gametogenesis in humans.

## Figures and Tables

**Table 1:** Information on all chemicals, antibodies, and recombinant proteins.

Name	Cat. No	Supplier
mTeSR1 Complete Kit	85850	Stemcell Technologies, Vancouver, BC, Canada
DMEM/F-12, no glutamine	21331020	Thermo Scientific, Waltham, MA, USA
KnockOut serum replacement	10828010	Gibco, Grand Island, NY, USA
Nonessential amino acid (NEAA)	11140035	Gibco, Grand Island, NY, USA
Glutamax	35050038	Gibco, Grand Island, NY, USA
Sodium pyruvate	1160039	Gibco, Grand Island, NY, USA
β-Mercaptoethanol	M6250	Sigma, St. Louis, MO, USA
TrypLe Express Enzyme (1×)	12604013	Thermo Scientific, Waltham, MA, USA
Rock inhibitor Y-27632 (ROCKi)	72304	Stemcell Technologies, Vancouver, BC, Canada
Accutase	07920	Stemcell Technologies, Vancouver, BC, Canada
1 U/mL dispase in DMEM/F-12	07923	Stemcell Technologies, Vancouver, BC, Canada
Recombinant human Activin A (rhActivinA)	PHC9564	Gibco, Grand Island, NY, USA
recombinant human BMP 4	PHC9534	Gibco, Grand Island, NY, USA
Corning^®^ Matrigel^®^ hESC-Qualified Matrix, LDEV-free, 5 mL	354277	CORNING, Corning, New York, USA
Recombinant human leukemia inhibitory factor	PHC9481	Gibco, Grand Island, NY, USA
DAPI	D9542	Sigma, St. Louis, MO, USA
Paraformaldehyde solution (4% (wt/vol)), buffered, pH 6.9	1004960700	Sigma, St. Louis, MO, USA
Optimal cutting temperature embedding cryo-embedding matrix (O.C.T.)	4583	Sakura, Torrance, CA, USA
Triton X-100	X100-5ML	Sigma, St. Louis, MO, USA
Tween 20	P2287-100ML	Sigma, St. Louis, MO, USA
FBS	100-0179	Stemcell Technologies, Vancouver, BC, Canada

**Table 2:** Information on all antibodies.

Antigens	Host	Cat. No	Source	Dilution
	Primary antibodies			
OCT4	Mouse	MAB4419	Millipore, Merck KGaA, Darmstadt, Germany	1:100
SOX2	Mouse	4900	Cell Signaling, Danvers, MA, USA	1:100
NANOG	Rabbit	ab21624	Abcam, Cambridge, United Kingdom	1:100
DAZL	Rabbit	PA5-54034	Fisher, Waltham, MA, USA	1:100
STELLA	Rabbit	PA5-34601	Fisher, Waltham, MA, USA	1:100
SOX17	Rabbit	PA5-72230	Fisher, Waltham, MA, USA	1:100
PRDM1	Mouse	MA5-38608	Fisher, Waltham, MA, USA	1:100
NANOS3	Rabbit	MA570093	Fisher, Waltham, MA, USA	1:100
SSEA1	Mouse	11-0159-42	Fisher, Waltham, MA, USA	1:100
cKIT	Rabbit	ZRB3201-25UL	Sigma, St. Louis, MO, USA	1:100
E-Cadherin	Rat	16-3249-82	Invitrogen, Carlsbad, CA, USA	1:100
	Secondary antibodies			
Alexa-488	Anti-mouse	A-21121	Life technologies, Carlsbad, CA, USA	1:1000
Texas Red	Anti-rabbit	TI-1000-1.5	Vector laboratories, Newark, CA, USA	1:1000
Alexa-488	Anti-rat	A-11006	Life technologies, Carlsbad, CA, USA	1:1000
**Genes**	**Primer Sequences (5′ to 3′)**	**Amplicon Size**		
*SSEA1*	F: AGGGGGTTCTTCCTCACCTT	107		
	R: ATATGGCCTGTGGCAGATGG			
*NANOS3*	F: GCTACACCTCCGTCTACAGC	133		
	R: CCGGCACCTCTGAAACCTG			
*PRDM14*	F: CTCTACGATCTGCCCTGGT	230		
	R: CTCAGCCCCTCAGGTAACAG			
*GAPDH*	F: TGCACCACCAACTGCTTAGC	87		
	R: GGCATGGACTGTGGTCATGAG			
*OCT4*	F: ACCCACACTGCAGCAGATCA	69		
	R: CACACTCGGACCACATCCTTCT			
*SOX2*	F: TGCGAGCGCTGCACAT	93		
	R: GCAGCGTGTACTTATCCTTCTTCA			
*NANOG*	F: ACAACTGGCCGAAGAATAGCA	63		
	R: GGTTCCCAGTCGGGTTCAC			
*KLF4*	F: ACCAGGCACTACCGTAAACACA	79		
	R: GGTCCGACCTGGAAAATGCT			
*PRDM1*	F: AAGCAACTGGATGCGCTATGT	108		
	R: GGGATGGGCTTAATGGTGTAGAA			
*DAZL*	F: ATGTTGTACCTCCGGCTTATTCA	118		
	R: CCATTTCCAGAGGGTGGAGTA			
*DNMT3B*	F: TGGAGCCACGACGTAACAAA	88		
	R: GCATCCGTCATCTTTCAGCCTA			
*PIWIL1*	F: GCATCAATGAAGGGATGACC	87		
	R: GGCAGACTTTGAGCCCATC			
*PIWIL4*	F: AGTGGAAGAGCCCGAGTGA	101		
	R: AGATCAACAGATCTAGGCAATGG			
*cKIT*	F: GCTTACCAGAAGCTTCCATAGTG	72		
	R: AGGTGAACTGCAAATACATAGGG			
*DDX4 (VASA)*	F: AGCTGGGACATTCAATTCGAC	220		

**Table 3:** Information on all consumables and equipment.

Name	Cat. No	Supplier
Micropipette set	F167350	Gilson, Middleton, WI, USA
5% CO_2_, 5% O_2_ humidified incubator set at 37 °C	MCO-19M	Sanyo, Osaka, Japan
Six-well clear tissue culture (TC)-treated multiple-well plates	10578911	Corning Costar, NY, USA
12-well clear TC-treated multiple-well plates	10253041	Corning Costar, NY, USA
Ultra-low-attachment multiple-well plate, size: 96 wells, round bottom, clear	7007	Corning Costar, NY, USA
Ultra-low-attachment Petri dish	3261 (60 mm)	Corning Costar, NY, USA
1.5 mL centrifuge tubes	LMTB015	GenFollower, Shaoxing City, Zhejiang Province (Shangyu District), China
15 mL centrifuge tubes	CFT511150	Jet Bio-Filtration, Guangzhou, Guangdong, China
LUNA automated cell counter	L10001-LG	Logos Biosystems, Anyang-si, Gyeonggi-do, South Korea
LUNA cell-counting slides	L12001	Logos Biosystems, Anyang-si, Gyeonggi-do, South Korea
40-μm cell strainer	93040	SPL, South Korea, South Korea
Zeiss Axio Vert.A1 Phase-contrast and fluorescence microscope		Zeiss, Oberkochen, Germany
Leica CM3050S Cryostat	CM3050 S	Leica, Wetzlar, Germany
Superfrost Plus slides	H867.1	
Coverslips, 24 × 50 mm	10212450C	
Vectashield mounting medium containing DAPI	H-1200-10	
ZEISS LSM 880 Confocal microscopy, Zeiss AxioImager D1 microscope equipped with an Axiocam HRm CCD camera		Carl Zeiss, Oberkochen, Germany
S3 Cell Sorter	145-1001	BioRad, Hercules, CA, USA

**Table 4:** Time planning.

Part	Hands-On Time	Incubation Time	Total Duration
Part A. hPSC maintenance and passaging	~20–30 min	Matrigel coating: 15–45 min; Dispase/TrypLE: 3–4 min; Cell attachment: 16–18 h	~16.5–19 h
Part B. hPGCLC differentiation in adhesive 2D culture	~20–30 min/day (daily medium changes and setup); additional ~1.5–2 h for FACS sample preparation	Activin A induction: 36–42 h; BMP4/LIF induction: 138–144 h; SSEA1 primary antibody: overnight (~16 h); Secondary antibody: 1.5 h; Cell dissociation: 3–7 min	~8 days (excluding flow cytometry analysis time)
Part C. EB generation and hPGCLC differentiation in embryoid bodies	~60–90 min for EB generation; ~10–20 min/day for medium changes	TrypLE: 3 min; EB aggregation: 16–18 h; Activin A induction: 36–42 h; BMP4/LIF induction: 138–144 h	~9 days

**Table 5:** Optional detailed timeline.

Stage	Duration
Part A: Matrigel coating	15–45 min
Cell passaging and plating	20–30 min hands-on
Cell attachment	16–18 h
Part B: Activin A induction	36–42 h (2 days)
BMP4/LIF induction	138–144 h (6 days)
Optional FACS staining	Overnight primary + 1.5 h secondary + 30–60 min sample preparation
Part C: Single-cell preparation and EB generation	60–90 min
EB aggregation	16–18 h
Activin A induction	36–42 h (2 days)
BMP4/LIF induction	138–144 h (6 days)

## Data Availability

The original contributions presented in this study are included in the article/Supplementary Material. Further inquiries can be directed to the corresponding author(s).

## Supplementary Materials

The following supporting information can be downloaded at: https://www.mdpi.com/article/10.3390/biology15161436/s1; Video S1: Time-lapse.Supp.video.

## Abbreviations

The following abbreviations are used in this manuscript:

Activin A	Recombinant human Activin A (rhActivinA)
ART	Assisted reproductive technologies
BMP4	Bone Morphogenetic Protein 4
DK15	hPGCLC basal medium—DK15
EB	Embryoid body
FACS	Fluorescence-activated cell sorting
hESCs	Human embryonic stem cells
hIPSCs	Human induced pluripotent stem cells
hPGCs	Human primordial germ cells
hPGCLCs	Human primordial germ cell-like cells
hPSCs	Human pluripotent stem cells
KSR	Knockout serum replacement
LIF	Leukemia inhibitory factor
NNEA	Nonessential amino acid
O.C.T.	Optimal cutting temperature embedding cryo-embedding matrix
RA	Retinoic acid
ROCKi	Rock inhibitor Y-27632
TC	Tissue culture
